# Defining Powerhouse Fruits and Vegetables: A Nutrient Density Approach

**DOI:** 10.5888/pcd11.130390

**Published:** 2014-06-05

**Authors:** Jennifer Di Noia

## Abstract

National nutrition guidelines emphasize consumption of powerhouse fruits and vegetables (PFV), foods most strongly associated with reduced chronic disease risk; yet efforts to define PFV are lacking. This study developed and validated a classification scheme defining PFV as foods providing, on average, 10% or more daily value per 100 kcal of 17 qualifying nutrients. Of 47 foods studied, 41 satisfied the powerhouse criterion and were more nutrient-dense than were non-PFV, providing preliminary evidence of the validity of the classification scheme. The proposed classification scheme is offered as a tool for nutrition education and dietary guidance.

## Objective

Powerhouse fruits and vegetables (PFV), foods most strongly associated with reduced chronic disease risk, are described as green leafy, yellow/orange, citrus, and cruciferous items, but a clear definition of PFV is lacking (1). Defining PFV on the basis of nutrient and phytochemical constituents is suggested (1). However, uniform data on food phytochemicals and corresponding intake recommendations are lacking (2). This article describes a classification scheme defining PFV on the basis of 17 nutrients of public health importance per the Food and Agriculture Organization of the United Nations and Institute of Medicine (ie, potassium, fiber, protein, calcium, iron, thiamin, riboflavin, niacin, folate, zinc, and vitamins A, B_6_, B_12_, C, D, E, and K) (3).

## Methods

This cross-sectional study identified PFV in a 3-step process. First, a tentative list of PFV consisting of green leafy, yellow/orange, citrus, and cruciferous items was generated on the basis of scientific literature (4,5) and consumer guidelines (6,7). Berry fruits and allium vegetables were added in light of their associations with reduced risks for cardiovascular and neurodegenerative diseases and some cancers (8). For each, and for 4 items (apples, bananas, corn, and potatoes) described elsewhere as low-nutrient-dense (1), information was collected in February 2014 on amounts of the 17 nutrients and kilocalories per 100 g of food (9). Because preparation methods can alter the nutrient content of foods (2), nutrient data were for the items in raw form.

Second, a nutrient density score was calculated for each food using the method of Darmon et al (10). The numerator is a nutrient adequacy score calculated as the mean of percent daily values (DVs) for the qualifying nutrients (based on a 2,000 kcal/d diet [11]) per 100 g of food. The scores were weighted using available data (Table 1) based on the bioavailability of the nutrients (12): nutrient adequacy score = (Σ [nutrient_i_ × bioavailability_i_)/DV_i_] × 100)/17. As some foods are excellent sources of a particular nutrient but contain few other nutrients, percent DVs were capped at 100 so that any one nutrient would not contribute unduly to the total score (3). The denominator is the energy density of the food (kilocalories per 100 g): nutrient density score (expressed per 100 kcal) = (nutrient adequacy score/energy density) x 100. The score represents the mean of percent DVs per 100 kcal of food.

**Table 1:** Bioavailability of Nutrients^a^ Used to Weight Nutrient Density Scores, 2014

Nutrient	Bioavailability, %
Iron	18
Riboflavin	95
Niacin	30
Folate	50
Vitamin B_6_	75
Vitamin B_12_	50
Vitamin C	70–90
Vitamin K	20

a Values shown represent the bioavailability of naturally occurring forms of the nutrients. When a range of values was reported, the lowest value in the range was used as the weighting factor.

Third, nutrient-dense foods (defined as those with scores ≥10) were classified as PFV. The Food and Drug Administration defines foods providing 10% or more DV of a nutrient as good sources of the nutrient (3). Because there are no standards defining good sources of a combination of nutrients-per-kilocalories, the FDA threshold was used for this purpose. The 4 low-nutrient-dense items were classified as non-PFV.

To validate the classification scheme, the Spearman correlation between nutrient density scores and powerhouse group was examined. The robustness of the scheme with respect to nutrients beneficial in chronic disease risk also was examined by comparing foods classified as PFV with those separately classified as such based on densities of 8 nutrients protective against cancer and heart disease (ie, fiber, folate, zinc, and vitamins B_6_, B_12_, C, D, and E) (2,4).

## Results

Of 47 foods studied, all but 6 (raspberry, tangerine, cranberry, garlic, onion, and blueberry) satisfied the powerhouse criterion (Table 2). Nutrient density scores ranged from 10.47 to 122.68 (median score = 32.23) and were moderately correlated with powerhouse group (ρ = 0.49, *P* = .001). The classification scheme was robust with respect to nutrients protective against chronic disease (97% of foods classified as PFV were separately classified as such on the basis of 8 nutrients protective against cancer and heart disease). For ease of interpretation, scores above 100 were capped at 100 (indicating that the food provides, on average, 100% DV of the qualifying nutrients per 100 kcal). Items in cruciferous (watercress, Chinese cabbage, collard green, kale, arugula) and green leafy (chard, beet green, spinach, chicory, leaf lettuce) groups were concentrated in the top half of the distribution of scores (Table 2) whereas items belonging to yellow/orange (carrot, tomato, winter squash, sweet potato), allium (scallion, leek), citrus (lemon, orange, lime, grapefruit), and berry (strawberry, blackberry) groups were concentrated in the bottom half (4–7).

**Table 2:** Powerhouse Fruits and Vegetables (N = 41), by Ranking of Nutrient Density Scores^a^, 2014

Item	Nutrient Density Score
Watercress	100.00
Chinese cabbage	91.99
Chard	89.27
Beet green	87.08
Spinach	86.43
Chicory	73.36
Leaf lettuce	70.73
Parsley	65.59
Romaine lettuce	63.48
Collard green	62.49
Turnip green	62.12
Mustard green	61.39
Endive	60.44
Chive	54.80
Kale	49.07
Dandelion green	46.34
Red pepper	41.26
Arugula	37.65
Broccoli	34.89
Pumpkin	33.82
Brussels sprout	32.23
Scallion	27.35
Kohlrabi	25.92
Cauliflower	25.13
Cabbage	24.51
Carrot	22.60
Tomato	20.37
Lemon	18.72
Iceberg lettuce	18.28
Strawberry	17.59
Radish	16.91
Winter squash (all varieties)	13.89
Orange	12.91
Lime	12.23
Grapefruit (pink and red)	11.64
Rutabaga	11.58
Turnip	11.43
Blackberry	11.39
Leek	10.69
Sweet potato	10.51
Grapefruit (white)	10.47

a Calculated as the mean of percent daily values (DVs) (based on a 2,000 kcal/d diet) for 17 nutrients (potassium, fiber, protein, calcium, iron, thiamin, riboflavin, niacin, folate, zinc, and vitamins A, B_6_, B_12_, C, D, E, and K) as provided by 100 g of food, expressed per 100 kcal of food. Scores above 100 were capped at 100 (indicating that the food provides, on average, 100% DV of the qualifying nutrients per 100 kcal).

## Discussion

The proposed classification scheme is offered in response to the call to better define PFV and may aid in strengthening the powerhouse message to the public. The focus on individual foods in terms of the nutrients they provide may facilitate better understanding of PFV than green leafy, yellow/orange, citrus, and cruciferous food groups that are emphasized. Messages might specify PFV to help consumers know what they are and choose them as part of their overall fruit and vegetable intake. As numeric descriptors of the amount of beneficial nutrients PFV contain relative to the energy they provide, the scores can serve as a platform for educating people on the concept of nutrient density. Expressing the nutrient desirability of foods in terms of the energy they provide may help focus consumers on their daily energy needs and getting the most nutrients from their foods. The rankings provide clarity on the nutrient quality of the different foods and may aid in the selection of more nutrient-dense items within the powerhouse group.

Foods within particular groups were studied; thus, other nutrient-dense items may have been overlooked. Because it was not possible to include phytochemical data in the calculation of nutrient density scores, the scores do not reflect all of the constituents that may confer health benefits. Warranting study is the utility of approaches defining PFV based on the presence (regardless of amount) of nutrients and phytochemicals. Although nutrient density differences by powerhouse group were examined, a true validation of the classification scheme is needed. Future studies might identify healthful diets and examine correlations with PFV or look for correlations between intake of PFV and health outcomes (3).

This study is an important step toward defining PFV and quantifying nutrient density differences among them. On the basis of the qualifying nutrients, 41 PFV were identified. The included foods may aid in improving consumer understanding of PFV and the beneficial nutrients they provide.

## References

[1] Nanney MS , Haire-Joshu D , Hessler K , Brownson RC . Rationale for a consistent “powerhouse” approach to vegetable and fruit messages. J Am Diet Assoc 2004;104(3):352–6. 10.1016/j.jada.2003.12.015 14993856

[2] World Cancer Research Fund. Food, nutrition, physical activity, and the prevention of cancer: a global perspective. Washington (DC): American Institute for Cancer Research; 2007.

[3] Drewnowski A . Concept of a nutritious food: toward a nutrient density score. Am J Clin Nutr 2005;82(4):721–32. 16210699 10.1093/ajcn/82.4.721

[4] Van Duyn MA , Pivonka E . Overview of the health benefits of fruit and vegetable consumption for the dietetics professional: selected literature. J Am Diet Assoc 2000;100(12):1511–21. 10.1016/S0002-8223(00)00420-X 11138444

[5] Higdon JV , Delage B , Williams DE , Dashwood RH . Cruciferous vegetables and human cancer risk: epidemiologic evidence and mechanistic basis. Pharmacol Res 2007;55(3):224–36. 10.1016/j.phrs.2007.01.009 17317210 PMC2737735

[6] Dietary Guidelines Advisory Committee. Report of the Dietary Guidelines Advisory Committee on the Dietary Guidelines for Americans, 2010, to the Secretary of Agriculture and the Secretary of Health and Human Services. Washington (DC): US Department of Agriculture, Agricultural Research Service; 2010.

[7] Shaw A , Fulton L , Davis C , Hogbin M . Using the food guide pyramid: a resource for nutrition educators. Alexandria (VA): US Department of Agriculture, Food, Nutrition, and Consumer Services, Center for Nutrition Policy and Promotion; 2001.

[8] Seeram NP . Recent trends and advances in berry health benefits research. J Agric Food Chem 2010;58(7):3869–70. 10.1021/jf902806j 20020687

[9] USDA national nutrient database for standard reference, release 26. Washington (DC): US Department of Agriculture, Agricultural Research Service; 2013.

[10] Darmon N , Darmon M , Maillot M , Drewnowski A . A nutrient density standard for vegetables and fruits: nutrients per calorie and nutrients per unit cost. J Am Diet Assoc 2005;105(12):1881–7. 10.1016/j.jada.2005.09.005 16321593

[11] A food labeling guide: guidance for industry. College Park (MD): Food and Drug Administration; 2013. http://www.fda.gov/downloads/Food/GuidanceRegulation/UCM265446.pdf. Accessed February 12, 2013.

[12] Otten JJ , Hellwig JP , Meyers LD , editors. Dietary reference intakes: the essential guide to nutrient requirements. Washington (DC): National Academies Press; 2006.

